# Transcription factor 3 (TCF3) combined with histone deacetylase 3 (HDAC3) down-regulates microRNA-101 to promote Burkitt lymphoma cell proliferation and inhibit apoptosis

**DOI:** 10.1080/21655979.2021.1977557

**Published:** 2021-10-17

**Authors:** Lihua Dong, Jingjing Huang, Peng Zu, Jing Liu, Xue Gao, Jianwei Du, Yufu Li

**Affiliations:** Department of Hematology, Henan Institute of Hematology, the Affiliated Cancer Hospital of Zhengzhou University, Zhengzhou, Henan Province, China

**Keywords:** Burkitt lymphoma, transcription factor TCF3, HDAC3, miR-101

## Abstract

To explore the function of transcription factor 3 (TCF3) on the proliferation and apoptosis of Burkitt lymphoma cells and its mechanism. qRT-PCR was performed to determine the expression of TCF3, histone deacetylase 3 (HDAC3), and microRNA-101 (miR-101) in the Burkitt lymphoma (BL) tumor tissues and lymph node tissues with reactive lymph node hyperplasia (RLNH). We found that the expression of TCF3 and HDAC3 was up-regulated in BL tumor tissues and lymphoma cells, and the miR-101 expression was down-regulated. And TCF3 and HDAC3 were negatively correlated with the expression of miR-101, respectively. In addition, knockdown of TCF3 can inhibit BL cell proliferation, reduce cell viability and promote cell apoptosis, retain the cell cycle in the G0/G1 phase, and inhibit the expression of Akt/mTOR pathway-related proteins (p-Akt and p-mTOR). When miR-101 was overexpressed, the results were the same as when TCF3 was knocked down. Moreover, we used Co-immunoprecipitation (Co-IP) to detect the interaction between TCF3 and HDAC3, and performed the Chromatin immunoprecipitation (ChIP) experiment to detect the enrichment of TCF3 and HDAC3 in the promoter region of miR-101. We found that TCF3 can interact with HDAC3 and is enriched in the miR-101 promoter region. In conclusion, TCF3 combined with HDAC3 down-regulates the expression of miR-101, thereby promoting the proliferation of BL cells and inhibiting their apoptosis.

## Introduction

Burkitt lymphoma (BL) is a highly aggressive non-Hodgkin’s B-cell lymphoma with endemic, sporadic, and immunodeficiency characteristics [1]. Currently, three Burkitt lymphoma cell subtypes have been found, which are endemic Burkitt lymphoma (eBL), sporadic Burkitt lymphoma (sBL), and immunodeficiency-related Burkitt lymphoma [2]. BL is one of the fastest-growing types of human tumors, and the time for cells to multiply is about 24 h [3]. Given the rapid progression of BL disease, biopsy and diagnosis should be accelerated. Currently, baseline examinations such as imaging and laboratory evaluation are mainly used to evaluate suspected BL disease [4]. BL mainly occurs in children and young people, and patients usually appear to have large tumors with high mortality [5]. At present, the treatment of BL mainly include short-term or long-term chemotherapy, using intensive drugs consisting of doxorubicin, alkylating agents, vincristine, and etoposide [6], but the effect is not ideal, most patients will undergo the treatment resistance, which making BL become a refractory disease [7]. In recent years, with the development of genomics technology, research has discovered the pathogenesis of several Burkitt lymphomas, among which the changes in the expression of the transcription factor 3 (TCF-3) (E2A) and its negative regulatory factor ID3 are the important induce factors to change the biological functions of Burkitt lymphoma cells [8]. A study has pointed out that TCF-3 gene expression has been found to be up-regulated in all three subtypes of Burkitt lymphoma [9]. However, there are few studies on the mechanism of how the transcription factor TCF-3 mediates the occurrence and development of Burkitt lymphoma. Therefore, to better prevent and treat Burkitt lymphoma, a more in-depth investigation is needed on this basis.

The activation of proto-oncogenes is usually accompanied by the inactivation of tumor suppressor genes, which is normally seen in the transcription and reprogramming of the tumor cells, affecting cell proliferation and cell function [10]. It has been found that histone regulation is essential in DNA damage control and gene transcription [11]. Histone deacetylases (HDACs) are widely distributed in the nucleus and are critical in regulating various pathophysiological processes (including metabolism, mitosis, apoptosis, and tumorigenesis) [12]. It has been reported that the HDAC3 function change is related to the occurrence of many cancers, including liver cancer [13], prostate cancer [14], rectal cancer [15], etc. At the same time, a study has pointed out that the recruitment of HDAC3 is linked with the maintenance of Burkitt lymphoma cell function [2], from which one can know that HDAC3 is related to the biological functions of tumor cells. On the other hand, in tumors, since the abnormal expression of microRNA (miRNA) is related to the development and metastasis of the disease, and treatment response as well as the overall survival of patients, miRNA is considered to be an important signaling molecule [16]. As early as 2002, there was a relevant report about miRNAs in lymphoid malignancies [17]. In recent years, studies have found that miR-101 is a type of tumor suppressor gene, which can inhibit the biological functions of colon cancer cells [18], prostate cancer [19], lung cancer cells [20], and other tumor cells. In addition, Ferreira et al [21] found that miR-101 was expressed at a lower level in the lymph node tissue of BL patients. However, it is not clear about the effect of miR-101 on the biological function of BL cells. Therefore, this study explored the effects of transcription factors TCF3 and miR-101 on the biological functions of BL cells *in vitro* and the determined the relationship between TCF3, HDAC3, and miR-101. This study aims to reveal the effect of TCF3 on the pathogenesis of Burkitt lymphoma and provide a reliable basis for clinical treatment.

## Materials and methods

### Tissue sample

We collected the tumor tissues from BL patients who were diagnosed and treated in our hospital from June 2018 to December 2020, and lymph node tissues from patients with reactive lymph node hyperplasia (RLNH) were collected during the same period. All patients signed an informed consent form about this study. This study was approved by the clinical trial ethics committee of our hospital and strictly followed the ‘Declaration of Helsinki’.

### Cell culture and transfection

Three BL cell lines (Namalwa, Raji, and Daudi cells) and human B lymphocyte cells (OCI-LY1) were all purchased from Shanghai Institutes for Biological Sciences (China), all of which were cultured in DMEM (Gibco, USA) medium containing 10% fetal bovine serum (FBS; Gibco, USA), and incubated at 37°C and 5% CO_2_.

We collected Namalwa, Raji, and Daudi cells in the logarithmic growth phase to prepare a single cell suspension and transfer it into a plate with 6 wells. Transfection was performed when the cells were cultured to a confluency of 50% to 60%. According to the operating instructions of Lipofectamine2000 (Invitrogen, USA), TCF3 siRNA (si-TCF3; 5′-AAGCAACAAAACATACACT-3′), HDAC3 siRNA (si-HDAC3; 5ʹ-GAUGCUGAACCAUGCACCUTT-3ʹ), miR-101 mimics (5ʹUACAGUACUGUGAUAACUGAA-3ʹ- and 5ʹ-UUCAGUUAUCACAGUACUGUA-3ʹ-), and negative controls (si-NC, NC mimics) were transfected into Namalwa, Raji, and Daudi cells, respectively, and then the plates were placed in a CO_2_ incubator at 37°C for 6 hours, then we checked the cell status, and continued to culture them for 24 hours. TCF3 siRNA, HDAC3 siRNA, miR-101 mimics, and the control sample were all synthesized by Guangzhou RiboBio Co., LTD (Guangzhou, China).

### qRT-PCR

The TRizol method was used to extract total RNA from tissues and cells, and a miRNA extraction and isolation kit (Tiangen, China) was used to extract miRNA. After detecting the concentration and purity of RNA, Random Primer Reverse Transcription Kit (Thermo, USA) was used to reversely transfer the mRNA to cDNA, and the miRNA cDNA first-strand synthesis kit (Tiangen, China) was used to synthesize the miRNA cDNA. The expression levels of TCF3, HDAC3, and miR-101 were detected following the instruction of the SYBR GREEN kit (TaKaRa, Japan). GAPDH and U6 were chosen as internal control controls, and the experiment set 6 replicates. The experimental data were dealt with the 2^−ΔΔCt^ method to calculate the relative expression of target genes. The primer sequences are in Table 1.

**Table 1.** Primer sequence

RNA	Sequences(5ʹ- to 3ʹ-)
TCF3	F: 5ʹ- CCAGACCAAACTGCTCATCCTG
	R: 5ʹ- TCGCCGTTTCAAACAGGCTGCT
miR-101	F: 5ʹ- GGGTGGCCATTGCTAATGCT
	R: 5ʹ-GCACTCAGGGTAGGTCAT
HDAC3	F: 5ʹ- GAGTTCTGCTCGCGTTACACAG
	R: 5ʹ- CGTTGACATAGCAGAAGCCAGAG
U6	F: 5ʹ- CTCGCTTCGGCAGCACAT
	R: 5ʹ- TTTGCGTGTCATCCTTGCG
GAPDH	F 5ʹ-CGGAGTCAACGGATTTGGTCGTAT-3’
	R 5ʹ-AGCCTTCTCCATGGTGGTGAAGAC-3’

### MTT experiment

The transfected cells were inoculated in a plate with 96 wells at a density of 5000 cells/well. After culturing for 24 h, 48 h, and 72 h respectively, 20 μL of 5 mg/mL MTT solution was added to each test well, and then the plates were continued to cultivate for 4 h in the incubator. Later, the supernatant was discarded, 150 μL of DMSO was added, and shaken for 15 minutes to measure the absorbance of each well with a wavelength of 570 nm using a microplate reader.

### Apoptosis detection

The transfected cells of each group were digested into centrifuge tubes by trypsin. The cells were washed twice with pre-cooled sterile PBS, and the cell concentration was adjusted to 5 × 10^5^ cells/mL. 200 μL of cell suspension was extracted to add with 10 μL of Annexin V-FITC, and then added with 10 μL of 20 mg/L PI solution, incubated for 10 min at room temperature in the dark. Later, 500 μL of PBS was added to detect the cell apoptosis through flow cytometry.

### Cell cycle detection

We collected the transfected cells of each group with a cell number of 1 × 10^6^ and rinsed the cells twice with pre-cooled sterile PBS. The cells were fixed with 70% ethanol at 4°C overnight. Later, the cells were washed twice with pre-chilled PBS and added PI solution to each well to incubate for 15 min in the dark at room temperature, and then the cell cycle was detected by flow cytometry.

### Co-immunoprecipitation (Co-IP)

After lysing the cells with RIPA lysate, we took the cell lysis of 1 mg/mL protein concentration to mix with the protein G beads (10003D, invitrogen, USA) at 4°C for 1.5 h to remove nonspecific binding. After centrifugation at 1000 g at 4°C, the supernatant was transferred to a new EP tube and incubated with IgG or target protein antibody TCF3 (Abcam, UK) at 4°C overnight. On the second day, 24 μL 50% protein G beads were added to incubate at 4°C for 2 h. The beads were washed for 5 times in the lysate, centrifuged at 2000 g at 4°C to discard the supernatant, added the same volume of the beads with 2 × SDS loading to heat at 100°C for 5 min and its liquid was collected, then the further Western blot experiment was performed.

### Chromatin immunoprecipitation (ChIP)

After treating the Namalwa cells with 4% formaldehyde for 10 min, they were washed with PBS three times, and SDS lysis buffer was added for lysis. After sonication, the primary antibody was added to incubate the precipitated protein-DNA complex. Then we used high-salt, low-salt, and LiCl buffer to wash, performed the elution buffer to collect the chromatin fragments, and amplified the DNA fragments co-precipitated with the protein by PCR. Finally, the PCR products were detected by agarose electrophoresis.

### Western blot

After transfecting for 24 h, the cells were lysed with cell lysate, and the total protein was extracted and the protein concentration was determined with the BCA kit (Thermo, USA). The protein was then separated by 10% SDS-PAGE and transferred to the PVDF membrane. After being blocked by 5% skimmed milk at room temperature for 1 h, the primary antibody TCF-3 (ab229605, Abcam, UK), Akt (ab38449, Abcam), p-Akt (ab18785, Abcam), mTOR (ab2732, Abcam), p-mTOR (ab109268, Abcam), and β-actin (No.66009–1, Proteintech, USA) were added and incubated overnight at 4°C. After washing the membrane 3 times, the secondary antibody was added and incubated for 1 h at room temperature. After washing the membrane another 3 times, a chemiluminescence reagent (Gibco, USA) was added to develop the protein and placed in the gel imaging system to collect the image. We employed Image J software to analyze the gray level of the protein band and selected β-actin as the internal reference to calculate the protein relative expression.

### Statistical analysis

The experimental data were analyzed by SPSS 26.0 software. The data following the normal distribution between the two groups were analyzed by an independent sample T-test. One-way analysis of variance was used for comparison between multiple groups. The results were expressed as mean ± standard deviation (SD). p < 0.05 was used as the criterion of the significance of the difference.

## Result

### Up-regulation of the TCF3 and down-regulation of the miR-101 in Burkitt lymphoma

**Figure 1.**
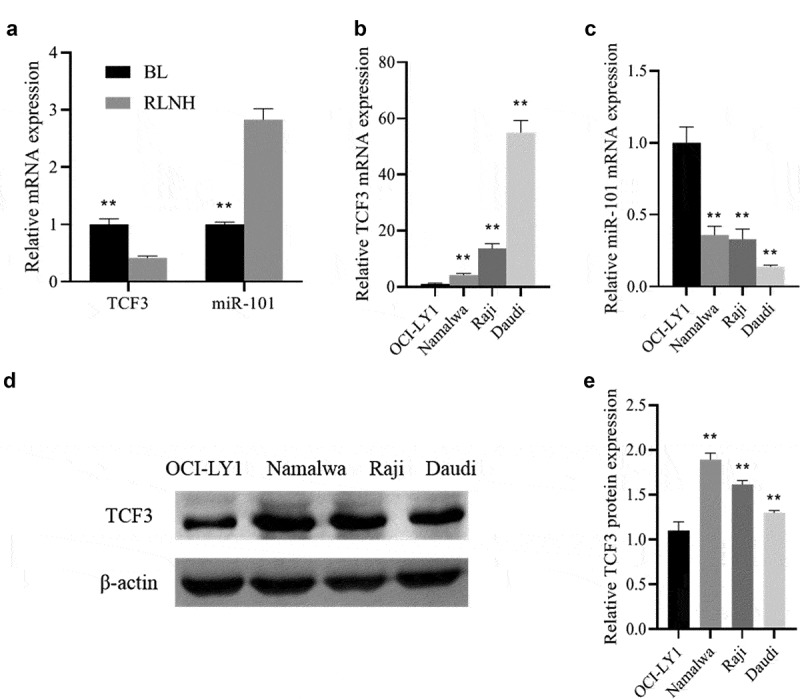
The expression of TCF3 and miR-101 in Burkitt lymphoma tissues and lymphoma cells

QRT-PCR was used to detect the expression of TCF3 and miR-101 in BL tissues and lymphoma cells. The results showed that when comparing to the RLNH tissue and lymphocyte OCI-LY1, the expression of TCF3 in BL tissues and lymphoma cells (Namalwa, Raji, Daudi) was dramatically higher, and the expression of miR-101 was notably lower (P < 0.01; Figure 1(a-c)). At the same time, the protein expression of TCF3 in Namalwa, Raji, and Daudi cells was also dramatically higher than that in OCI-LY1 cells (P < 0.01), with the highest expression in Namalwa cells and the lowest expression in Daudi cells (Figure 1(d-e)). These results showed that TCF3 and miR-101 may be involved in the occurrence of Burkitt lymphoma.

A: Determination of the expression of TCF3 and miR-101 in Burkitt lymphoma (BL) tissues and lymph node reactive hyperplasia tissues (RLNH) using qRT-PCR, **p < 0.01 vs. RLNH group; B: Determination of the TCF3 expression in the lymphoma cell lines (Namalwa, Raji, Daudi) and lymphocytes (OCI-LY1) using qRT-PCR; C: Determination of the expression of miR-101 using qRT-PCR; D: Evaluation of the expression of TCF3 protein using Western blot; E: Protein quantitative grayscale statistics, **p < 0.01 vs. OCI-LY1 group.

### Inhibition of the lymphoma cell proliferation and promotion of its apoptosis by TCF3 knockdown

**Figure 2.**
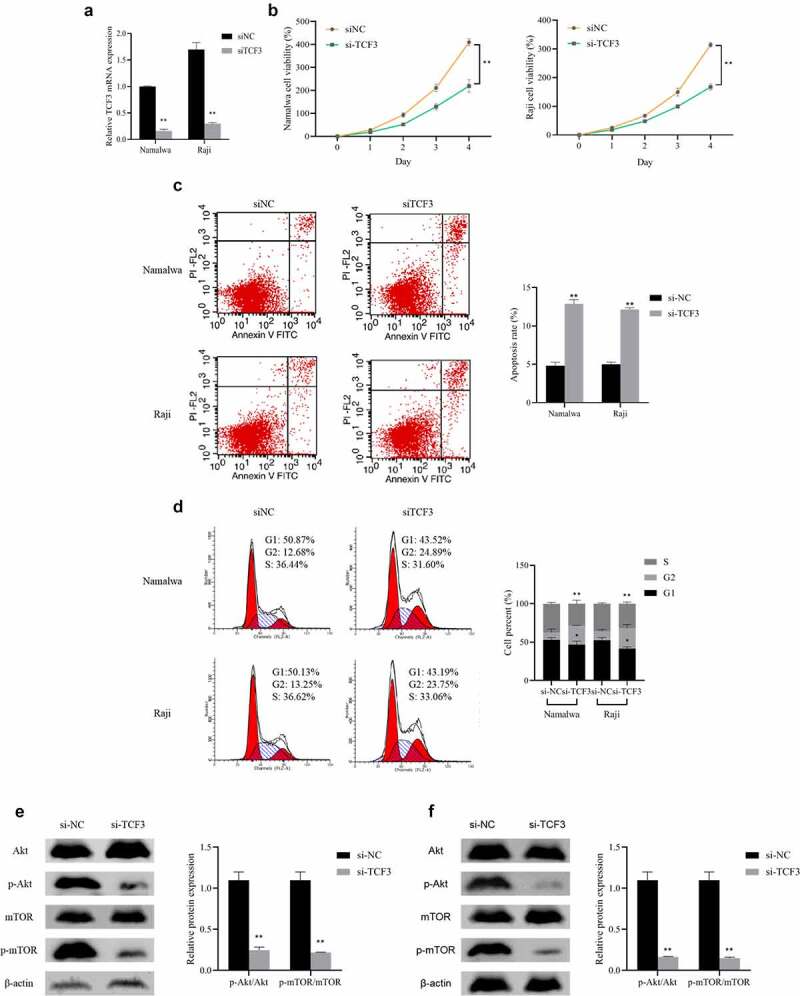
Influence of BL cell proliferation, apoptosis, cell cycle and Akt/mTOR pathway by knocking down TCF3

To further determine the function of TCF3 in Burkitt lymphoma, we knocked down TCF3 in Namalwa and Raji cells and tested its influence on cell proliferation, apoptosis, and cell cycle. First, qRT-PCR was used to verify the transfection efficiency of TCF3 siRNA in cells, which can notably reduce the expression of TCF3 in BL cells (P < 0.01; Figure 2(a)). Subsequently, after knocking down the TCF3, the proliferation rate of lymphoma cells was dramatically lower, while the apoptosis rate was distinctly higher (P < 0.01). And the proportion of cells in the G2 phase significantly increased (P < 0.01), indicating that the cells were blocked in the G2 phase and were unable to undergo mitosis (Figure 2(b-d)).

A study has shown that the expression of p-AKT and p-mTOR may be a potential reference and independent prognostic indicator for the diagnosis of pediatric BL [22]. Therefore, we interfered with the expression of TCF3 in Namalwa and Raji cells and detected the expression of p-Akt and p-mTOR. When comparing to the si-NC group, the ratios of p-Akt/Akt and p-mTOR/mTOR in BL cells were significantly decreased after knocking down TCF3 (P < 0.01; Figure 2(e-f)). These results indicated that interference with TCF3 expression can inhibit BL cell proliferation and cycle progression, promote cell apoptosis, and inhibit the activity of the Akt/mTOR pathway.

A: Evaluation of the expression of TCF3 in Namalwa and Raji cells after transfection of TCF3 siRNA using qRT-PCR; B: Assessment of the proliferation rate using MTT; C: Evaluation of cell apoptosis using flow cytometry; D: Determination of the cell cycle using flow cytometry; E: Assessment of the expression of Akt/mTOR pathway in Namalwa cells using Western blot; F: Determination of the expression of Akt/mTOR pathway in Raji cells using Western blot, **p < 0.01 vs. si-NC group.

### Inhibition of the lymphoma cell proliferation and promotion of the apoptosis by miR-101 over-expression

**Figure 3.**
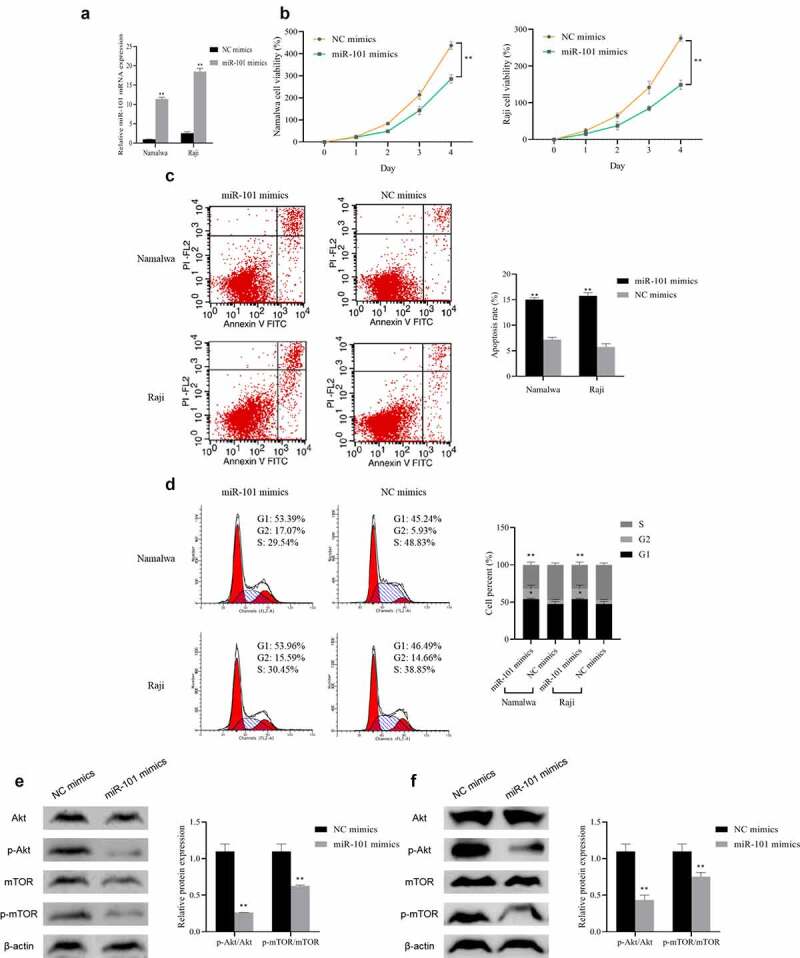
The effect of miR-101 on lymphoma cell proliferation, apoptosis, cell cycle and Akt/mTOR pathway

Furthermore, we detected the influence of miR-101 over-expression in Namalwa cells and Raji cells by overexpressing the miR-101. The qRT-PCR results showed that the expression of miR-101 in the cells transfected with miR-101 mimics was dramatically increased (P < 0.01), indicating that the overexpression of miR-101 was successful (Figure 3(a)). When comparing to the NC mimics group, miR-101 overexpression can significantly inhibit the proliferation of Namalwa and Raji cells and promote cell apoptosis, meanwhile, the proportion of cells in the G2 phase is notably higher, while the proportion of cells in the S phase is distinctly lower (P < 0.01; Figure 3(b-d)). And after overexpression of miR-101, the expression levels of Akt and mTOR in cells showed no obvious difference, while the ratios of p-Akt/Akt and p-mTOR/mTOR decreased distinctly (P < 0.01; Figure 3(e-f)). These results indicated that overexpression of miR-101 can inhibit BL cell proliferation and cycle progression, promote cell apoptosis, and suppress the activation of the Akt/mTOR pathway.

A: Detection of the expression of miR-101 in Namalwa and Raji cells transfected with miR-101 mimics using qRT-PCR; B: Assessment of the proliferation rate using MTT; C: Evaluation of the apoptosis using flow cytometry; D: Detection of the cell cycle using flow cytometry; E: Evaluation of the Akt/mTOR pathway protein in Namalwa cells using Western blot; F: Assessment of Akt/mTOR pathway protein expression in Raji cells using Western blot, **p < 0.01 vs. NC mimics group.

### Up-regulation of the miR-101 expression by knocking down HDAC3 or TCF3

**Figure 4.**
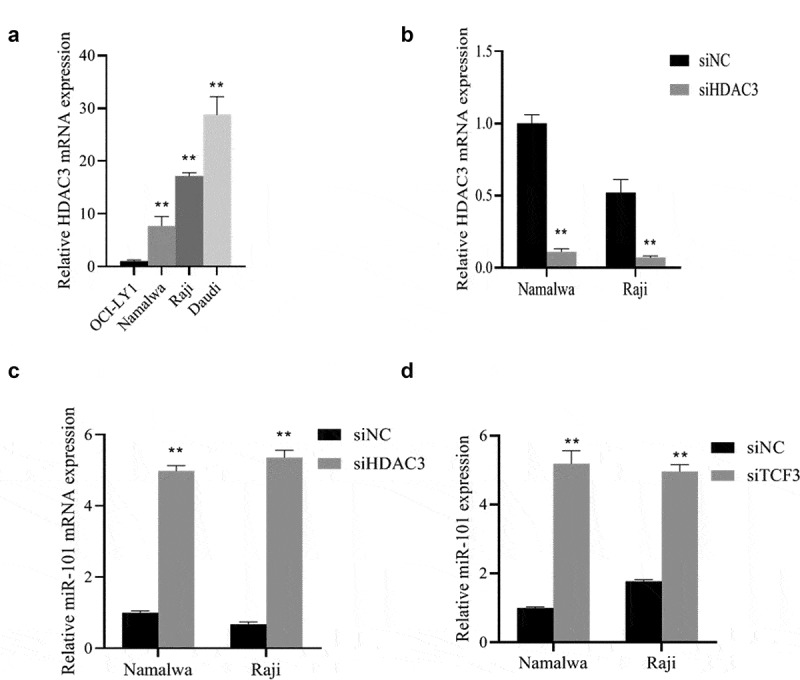
The expression level of miR-101 after knocking down HDAC3 and TCF3

After determining the functions of TCF3 and miR-101 in lymphoma cells, we further examined their molecular mechanisms. First, we used qRT-PCR to detect the expression of HDAC3 in Burkitt lymphoma cell lines OCI-LY1, Namalwa, Raji, and Daudi and found that the expression of HDAC3 in lymphoma cells was notably upregulated (P < 0.01), with the highest expression in Namalwa cells (Figure 4(a)). Subsequently, after interfering with HDAC3, its expression was distinctly decreased in the lymphoma cells Namalwa and Raji cells (P < 0.01; Figure 4(b)). After successful transfection, we found that knockdown of HDAC3 can up-regulate the expression of miR-101 in Namalwa and Raji cells, and knockdown of TCF3 can also up-regulate the expression of miR-101 (P < 0.01; Figure 4(c,d)), which showed that HDAC3 and TCF3 were negatively correlated with miR-101 expression in BL.

A: Determination of the expression of HDAC3 using qRT-PCR, **p < 0.01 vs. OCI-LY1 group; B: Evaluation of the expression of HDAC3 using qRT-PCR; C: Assessment of the expression level of miR-101 in cells after HDAC3 knockdown using qRT-PCR; E: Assessment of the expression level of miR-101 in cells after TCF3 knockdown using qRT-PCR; **p < 0.01 vs. si NC group.

### Both TCF3 and HDAC3 can bind to the miR-101 promoter

**Figure 5.**
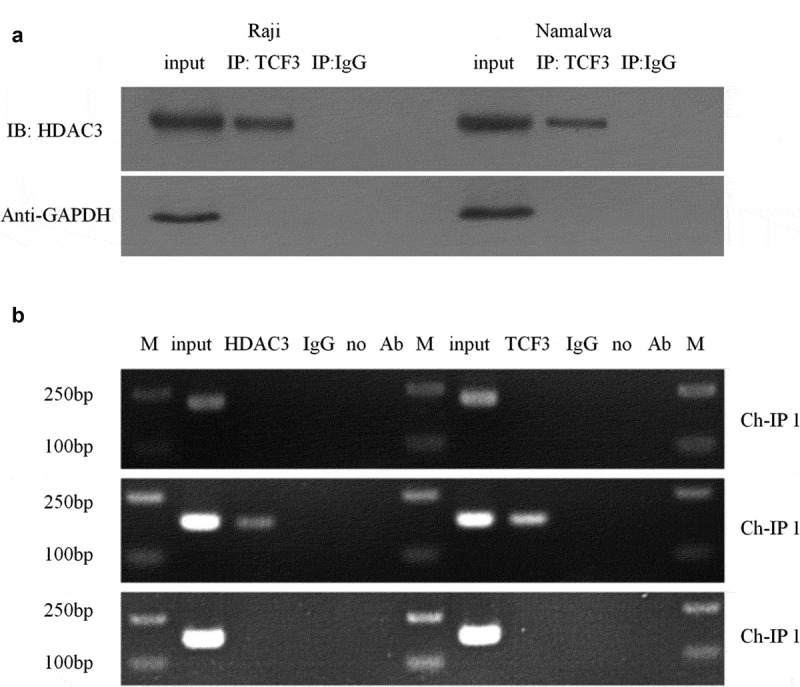
The relationship between TCF3, HDAC3 and miR-101

Histone acetylation will promote the specific binding of transcription factors to DNA binding sites during gene transcription, thereby activating gene transcription. Therefore, we used Co-IP to detect the interaction between HDAC3 and TCF3 in order to further study the relationship between HDAC3, TCF3, and miR-101. The results showed that TCF3 antibodies can bind to HDAC3 protein in Namalwa and Raji cells (Figure 5(a)). Furthermore, we performed ChIP experiments to determine the combination of HDAC3, TCF3, and miR-101 promoters. The results showed that TCF3 and HDAC3 can be enriched in the miR-101 promoter region (Figure 5(b)).

A: Verification of the interaction between HDAC3 and TCF3 in Namalwa and Raji cells using Co-IP; IP, immunoprecipitation; IB, immunoblotting; B Electropherogram of ChIP detection of TCF3 and HDAC3 enrichment in different promoter regions of miR-101; M, Marker; no Ab, no antibody.

## Discussion

The biological characteristic of tumor cells is their rapid proliferation, so it must have a corresponding anabolic rate to meet the requirements of its growth and development [23], so the cell metabolism change is one of the signs of tumor cells. Cancer metabolic reprogramming involves three basic changes, including changes in biological performance, energy utilization, and biosynthesis and redox balance efficiency, all of which together improve the adaptability of cells, thereby providing the selectivity advantage for tumors [24]. The cell biological performance change is the most direct change of tumor cells, including proliferation, migration, invasion, and so on. According to the other research, TCF3 has a basic helix-loop-helix (B-HLH) structure, and it can contact the main groove of DNA through its HLH domain homodimer and basic region, and then start the gene expression [25]. The accumulation of TCF3 is generally related to the growth and development of tumor cells [26], so TCF3 is considered to be a transcription factor that regulates tumor cell proliferation, migration, and invasion [9]. At the same time, studies have found that silencing the expression of TCF3 can greatly reduce the possibility of forming the tumor of breast cancer, leading to a decrease in tumor growth rate [27]. In this study, TCF3 was found to be up-regulated in Burkitt lymphoma tissues and cells, and when TCF3 was knocked down, it was observed that the proliferation rate and cell viability of BL cells were distinctly lower, and the apoptosis rate was notably higher, suggesting that the rapid growth and development of Burkitt lymphoma cells is related to the high expression of TCF3.

miR-101 has been confirmed to be closely related to cell proliferation, apoptosis, and cell cycle. It has been recognized that it can inhibit tumor proliferation by regulating several target genes [28], for example, miR-101 can directly inhibit cell proliferation by reducing the expression of zeste homolog 2 (EZH2) enhancer in lung cancer and embryonic rhabdomyosarcoma (eRMS) [29]. At the same time, it has been confirmed that miR-101 directly increases breast cancer cell apoptosis by reducing the expression of Janus kinase (Jak2) [30]. A study has also found that miR-101 can inhibit cell proliferation and promote cell apoptosis by regulating the phosphorylation of key proteins PI3K, PTEN, AKT, and mTOR in the Akt/mTOR pathway [31], which indicated that miR-101 is the gene that can inhibit tumor development. In this study, it was found that the expression of miR-101 in Burkitt lymphoma tissues and cells was down-regulated, but when the up-regulating its expression, the biological activities of Burkitt lymphoma cells, such as proliferation, cell viability, etc. and the related proteins of Akt/mTOR pathway were distinctly suppressed. In addition, the up-regulation of miR-101 after interference with TCF3 expression indicates that there is a relationship between these two. These results suggest that TCF3 may be involved in the pathogenesis of BL through increasing the miR-101 expression.

Further we explored the mechanisms by which TCF3 mediates BL disease development. It is well known that transcription factors need to recruit DNA modifying enzymes to function [32]. HDAC3, an important class of epigenetic regulation and modification enzymes, is widely present in the nucleus and essential in regulating various pathophysiological processes. It has been pointed out that the accumulation of HDAC3 is directly related to the promotion of tumor occurrence and development [33]. Cao et al. found that inhibiting HDAC3 can be used to inhibit the growth and development of tumor cells and has the potential to treat tumor-related diseases [34]. Therefore, this study firstly discovered the interaction between TCF3 and HDAC3 through the Co-IP experiment. Knocking down HDAC3 can also up-regulate the expression of miR-101, so there is a negative correlation between the expression of HDAC3 and miR-101 in Burkitt lymphoma cells. Further ChIP experiment revealed that TCF3 and HDAC3 could be enriched in the miR-101 promoter region. It is thus hypothesized that HDAC3 is the key mediator of TCF3 affecting the expression of miR-101. So far, it can be seen that the highly expressed transcription factor TCF3 in Burkitt lymphoma can recruit HDAC3 in the miR-101 promoter region, inhibit miR-101 expression, and regulate the activity of the Akt/mTOR pathway, thereby improving the biological performance of Burkitt lymphoma cells.

## Conclusion

In summary, TCF3 can bind to HDAC3 to downregulate miR-101 expression, which in turn regulates Akt/mTOR signaling pathway activity, thereby inhibiting the proliferation of BL cells and promoting cell apoptosis. This study suggests that TCF3 can be a biological marker of BL and may be a potential therapeutic target for this disease. However, the potential target genes that miR-101 may regulate in BL and its relationship with the clinicopathological features of BL need to be further explored.

## Highlight

1. The expression of TCF3 and HDAC3 was up-regulated in Burkitt lymphoma (BL), and the miR-101 expression was down-regulated.
2. Knockdown of TCF3 can inhibit BL cell proliferation and viability, but promote cell apoptosis.
3. Low expression of TCF3 inhibits Akt/mTOR pathway activity.
4. TCF3 combined with HDAC3 down-regulates the expression of miR-101.

## References

[1] Shapira J, Peylan-Ramu N. Burkitt’s lymphoma. Oral Oncol. 1998;34(1):15–23. 9659515 10.1016/s1368-8375(97)00041-9

[2] Dong LH, Cheng S, Zheng Z, et al. Histone deacetylase inhibitor potentiated the ability of MTOR inhibitor to induce autophagic cell death in Burkitt leukemia/lymphoma. J Hematol Oncol. 2013;6(1):53. 23866964 10.1186/1756-8722-6-53 PMC3722002

[3] Ferry JA. Burkitt’s lymphoma: clinicopathologic features and differential diagnosis. Oncologist. 2006;11(4):375–383. 16614233 10.1634/theoncologist.11-4-375

[4] Crombie J, LaCasce A. The treatment of Burkitt lymphoma in adults. Blood. 2021;137(6):743–750. 33171490 10.1182/blood.2019004099

[5] Dave SS, Fu K, Wright GW, et al. Molecular diagnosis of Burkitt’s lymphoma. N Engl J Med. 2006;354(23):2431–2442. 16760443 10.1056/NEJMoa055759

[6] Schmitz R, Ceribelli M, Pittaluga S, et al. Oncogenic mechanisms in Burkitt lymphoma. Cold Spring Harb Perspect Med. 2014;4(2):a14282. 10.1101/cshperspect.a014282 PMC3904095 24492847

[7] Heslop HE. Sensitizing Burkitt lymphoma to EBV-CTLs. Blood. 2020;135(21):1822–1823. 32437562 10.1182/blood.2020005492 PMC7243150

[8] Richter J, Schlesner M, Hoffmann S, et al. Recurrent mutation of the ID3 gene in Burkitt lymphoma identified by integrated genome, exome and transcriptome sequencing. Nat Genet. 2012;44:1316–1320. 23143595 10.1038/ng.2469

[9] Schmitz R, Young RM, Ceribelli M, et al. Burkitt lymphoma pathogenesis and therapeutic targets from structural and functional genomics. Nature. 2012;490(7418):116–120. 22885699 10.1038/nature11378 PMC3609867

[10] Raggi C, Factor VM, Seo D, et al. Epigenetic reprogramming modulates malignant properties of human liver cancer. Hepatology. 2014;59(6):2251–2262. 24449497 10.1002/hep.27026 PMC4043911

[11] Jenuwein T, Allis CD. Translating the histone code. Science. 2001;293(5532):1074–1080. 11498575 10.1126/science.1063127

[12] Gallinari P, Di Marco S, Jones P, et al. HDACs, histone deacetylation and gene transcription: from molecular biology to cancer therapeutics. Cell Res. 2007;17(3):195–211. 17325692 10.1038/sj.cr.7310149

[13] Ji H, Zhou Y, Zhuang X, et al. Correction: HDAC3 deficiency promotes liver cancer through a defect in H3K9ac/H3K9me3 transition. Cancer Res. 2020;80(4):923. 32060229 10.1158/0008-5472.CAN-19-3887

[14] Yan Y, An J, Yang Y, et al. Dual inhibition of AKT-mTOR and AR signaling by targeting HDAC3 in PTEN – or SPOP-mutated prostate cancer. EMBO Mol Med. 2018;10(4):1759–1768. 10.15252/emmm.201708478 PMC5887910 29523594

[15] Li J, Hu M, Liu N, et al. HDAC3 deteriorates colorectal cancer progression via microRNA-296-3p/TGIF1/TGFβ axis. J Exp Clin Cancer Res. 2020;39(1):248. 33203425 10.1186/s13046-020-01720-w PMC7670781

[16] Monroig-Bosque Pdel C, Rivera CA, Calin GA. MicroRNAs in cancer therapeutics: “from the bench to the bedside”. Expert Opin Biol Ther. 2015;15(10):1381–1385. 26372796 10.1517/14712598.2015.1074999 PMC4890620

[17] Bartel DP. MicroRNAs: genomics, biogenesis, mechanism, and function. Cell. 2004;116(2):281–297. 14744438 10.1016/s0092-8674(04)00045-5

[18] Huang Z, Wu X, Li J. miR-101 suppresses colon cancer cell migration through the regulation of EZH2. Rev Esp Enferm Dig. 2021;113:255–260. 33207890 10.17235/reed.2020.6800/2019

[19] Gu Z, You Z, Yang Y, et al. Inhibition of MicroRNA miR-101-3p on prostate cancer progression by regulating Cullin 4B (CUL4B) and PI3K/AKT/mTOR signaling pathways. Bioengineered. 2021;12(1):4719–4735. 34338146 10.1080/21655979.2021.1949513 PMC8806765

[20] Meng X, Sun Y, Liu S, et al. miR-101-3p sensitizes lung adenocarcinoma cells to irradiation via targeting BIRC5. Oncol Lett. 2021;21(4):282. 33732358 10.3892/ol.2021.12543 PMC7905603

[21] Ferreira AC, Robaina MC, Rezende LM, et al. Histone deacetylase inhibitor prevents cell growth in Burkitt’s lymphoma by regulating PI3K/Akt pathways and leads to upregulation of miR-143, miR-145, and miR-101. Ann Hematol. 2014;93:983–993. 24577510 10.1007/s00277-014-2021-4

[22] Man J, Chen L, Zhai XW, et al. Expression of p-AKT and p-mTOR in pediatric Burkitt lymphoma and their correlation with prognosis. Zhonghua Bing Li Xue Za Zhi Chinese Journal of Pathology. 2020;49:156–161. 32074729 10.3760/cma.j.issn.0529-5807.2020.02.010

[23] Dozzo M, Carobolante F, Donisi PM, et al. Burkitt lymphoma in adolescents and young adults: management challenges. Adolesc Health Med Ther. 2017;8:11–29. 28096698 10.2147/AHMT.S94170 PMC5207020

[24] Białopiotrowicz E, Noyszewska-Kania M, Kachamakova-Trojanowska N, et al. Serine biosynthesis pathway supports MYC-miR-494-EZH2 feed-forward circuit necessary to maintain metabolic and epigenetic reprogramming of Burkitt lymphoma cells. Cancers (Basel). 2020;12(3):580. 10.3390/cancers12030580 PMC7139810 32138178

[25] Shah M, Rennoll SA, Raup-Konsavage WM, et al. A dynamic exchange of TCF3 and TCF4 transcription factors controls MYC expression in colorectal cancer cells. Cell Cycle. 2015;14(3):323–332. 25659031 10.4161/15384101.2014.980643 PMC4353228

[26] Li L, Zheng YL, Jiang C, et al. HN1L-mediated transcriptional axis AP-2γ/METTL13/TCF3-ZEB1 drives tumor growth and metastasis in hepatocellular carcinoma. Cell Death Differ. 2019;26(11):2268–2283. 30778199 10.1038/s41418-019-0301-1 PMC6889153

[27] Slyper M, Shahar A, Bar-Ziv A, et al. Control of breast cancer growth and initiation by the stem cell-associated transcription factor TCF3. Cancer Res. 2012;72(21):5613–5624. 23090119 10.1158/0008-5472.CAN-12-0119

[28] Friedman JM, Liang G, Liu CC, et al. The putative tumor suppressor microRNA-101 modulates the cancer epigenome by repressing the polycomb group protein EZH2. Cancer Res. 2009;69(6):2623–2629. 19258506 10.1158/0008-5472.CAN-08-3114

[29] Zhang JG, Guo JF, Liu DL, et al. MicroRNA-101 exerts tumor-suppressive functions in non-small cell lung cancer through directly targeting enhancer of zeste homolog 2. J Thorac Oncol. 2011;6(4):671–678. 21270667 10.1097/JTO.0b013e318208eb35

[30] Wang L, Li L, Guo R, et al. miR-101 promotes breast cancer cell apoptosis by targeting Janus kinase 2. Cell Physiol Biochem. 2014;34(2):413–422. 25059472 10.1159/000363010

[31] An X, Ma H, Liu Y, et al. Effects of miR-101-3p on goat granulosa cells in vitro and ovarian development in vivo via STC1. J Anim Sci Biotechnol. 2020;11(1):102. 33072314 10.1186/s40104-020-00506-6 PMC7557009

[32] Dal BM, Bomben R, Hernández L, et al. The MYC/miR-17-92 axis in lymphoproliferative disorders: a common pathway with therapeutic potential. Oncotarget. 2015;6(23):19381–19392. 26305986 10.18632/oncotarget.4574 PMC4637292

[33] Hull EE, Montgomery MR, Leyva KJ. HDAC inhibitors as epigenetic regulators of the immune system: impacts on cancer therapy and inflammatory diseases. Biomed Res Int. 2016;2016:8797206. 27556043 10.1155/2016/8797206 PMC4983322

[34] Cao F, Zwinderman MRH, Dekker FJ. The process and strategy for developing selective histone deacetylase 3 inhibitors. Molecules. 2018;23(3):551. 10.3390/molecules23030551 PMC6017514 29498635

